# Examining the relationship between socio-economic status, WASH practices and wasting

**DOI:** 10.1371/journal.pone.0172134

**Published:** 2017-03-09

**Authors:** Mohammad Jyoti Raihan, Fahmida Dil Farzana, Sabiha Sultana, Md Ahshanul Haque, Ahmed Shafiqur Rahman, Jillian L. Waid, Ben McCormick, Nuzhat Choudhury, Tahmeed Ahmed

**Affiliations:** 1 Nutrition and Clinical Services Division, International Centre for Diarrhoeal Disease Research, Bangladesh, Dhaka, Bangladesh; 2 James P Grant School of Public Health, BRAC University, Dhaka, Bangladesh; 3 Helen Keller International, Dhaka, Bangladesh; 4 Fogarty International Center/National Institutes of Health, Bethesda, MD, United States of America; BRAC, BANGLADESH

## Abstract

Childhood wasting is a global problem and is significantly more pronounced in low and middle income countries like Bangladesh. Socio Economic Status (SES) and Water, Sanitation and Hygiene (WASH) practices may be significantly associated with wasting. Most previous research is consistent about the role of SES, but the significance of WASH in the context of wasting remains ambiguous. The effect of SES and WASH on weight for length (WHZ) is examined using a Structural Equation Model (SEM) to explicitly describe the direct and indirect role of WASH in the context of SES.A nationally representative survey of 10,478 Bangladeshi children under 5 were examined. An expert defined SEM was used to construct latent variables for SES and WASH. The SEM included a direct pathway from SES to WHZ and an indirect pathway from SES to WHZ via WASH along with regression of relevant covariates on the outcome WHZ and the latent variables. Both SES (p<0.01) and WASH (p<0.05) significantly affect WHZ. SES (p<0.01) also significantly affects WASH. Other structural components showed that child’s age (p<0.01) affects WHZ and types of residence (p<0.01) affects SES. WASH practices at least partially mediate the association between SES and wasting status. WASH and SES are both significantly associated with WHZ.

## Introduction

Wasting, as an indicator of acute malnutrition, is defined by a weight-for-height/length z-score (WHZ) of more than -2 standard deviations below the World Health Organization (WHO) growth standard [1]. Wasting represents thinness and signifies recent failure to gain optimal body weight or loss of body weight, which is often associated with persistent hostile environments, chronic disease or acute starvation [2–4]. Wasting is regarded as a leading contributor to under 5 child mortality and morbidity in low and middle income countries [5–7], with the case fatality rates for wasted children around 5–60% [8–10], in part because wasting acts as a precursor to common childhood illnesses such as diarrhea and pneumonia. Adverse effect on long term physical and cognitive development have also been suggested [8].

Though the prevalence of malnutrition in Asia is decreasing, Bangladesh and its neighbors still have the highest cumulative number of malnourished children [11–13]. The significant reduction in prevalence of stunted and underweight Bangladeshi children in the past 10 years, highlights the considerable effort of the government and other national and international stakeholders in nutrition and holistically portrays the success of the country’s health system. The stagnancy in the prevalence of wasting, which has continued at around 15%, a figure that the World Health Organization (WHO) class as ‘critical’ [3], may not then, be due to any systematic failure of the health system. Some assessment of the many intervention programs oriented towards the reduction of incidence and prevalence of wasting in the country along with the associated risk factors is then needed to understand the point at which continued improvement can be found.

Factors associated with wasting are diverse [4,14,15], but can be broadly categorized into proximal maternal and child functions and distal socio-economic determinants [16]. The socio-economic factors consist of resources needed for adequate food security, hygienic environment and optimal child care [15]. Despite having no general consensus among the scientific community about which indictors appropriately contribute to a composite socio-economic variable, cited literature suggested women’s education, access to safe water and sanitation and household economic status can be used as proxy indicators [15].

The effects of socio-economic status (SES) on WHZ is well documented [4,17]. Many studies have also identified associations between poor Water, Sanitation and Hygiene (WASH) practices with wasting [18–21], which is critical given that WASH is a practical intervention in the malnutrition disease cycle. Socio-economic disparity in context of optimal hygiene and WASH practices has been the salient finding of many studies [22–24]. Optimal WASH practices require improved education, infrastructure and communal improvements to water and sanitation, all of which is reflected by the socio-economic conditions of the households.

Many studies [12,25–29] have used variables related to safe water consumption, optimal sanitation and hygiene practices to construct a domain commonly referred as WASH. The critical role of optimal WASH practices for better health is well established [30–32] and with regards wasting in particular [13]. It should be noted that optimal WASH practices invariably includes the use of soap [33,34]. Handwashing using soap removes or kills transient flora present on the skin surface [35,36], for example bacteria such as *P*. *pyocyanea* and strains of *Salmonella*, *Shigella* and *E*. *coli* [37] that are all associated with diarrheal disease [38,39]. On other aspects of WASH, a study determining the factors associated with childhood malnutrition in 36 low and middle income countries suggested that the use of pit latrine and flush toilets have significantly positive effect on WHZ [15], which can also be linked to reduced rate of gut or enteric infections [32]. However, a comprehensive review which included analysis on the effect of improved water quality, sanitation and hygiene practices on WHZ of 4,322 under 5 children score found no such significant association [12]. Similar statistically non-significant associations between SES determinants and WHZ were observed in nationally representative samples of under 5 Bangladeshi children [14,17,32]. The inconsistent effect of WASH and SES on WHZ may be due to statistical methods used to measure and represent SES and WASH in relevant studies, for example the number and identity of other regressors included in analyses and the inherently overlapping variance explained by individual SES and WASH indicators. More importantly, for WASH interventions not to work effectively in resource poor settings, like that of Bangladesh, is the intervention components itself. Existing WASH interventions showed to hinder fecal-oral transmission of pathogens, which is responsible for causing undernutrition among children [29]. Common WASH interventions in Bangladesh failed to provide improved environmental hygiene for children as the interventions were not effective to moderate risky behaviors such as crawling and picking objects from contaminated soil and surfaces [29] and *pica* [40].

Therefore, to explicitly address the relationships between SES and WASH and their joint relationships with WHZ, a Structural Equation Model (SEM) using the causal mediation analysis approach [41,42] is presented. The SEM offers an integrated approach with increased validity as the construction of latent variables captures the multifactorial properties of inter-related domains of which no single observable variable would be representative [42,43].

To formally address the interrelationships of SES and WASH and their respective contribution to the prevalence of WHZ, we hypothesize that SES affects WHZ, but that SES is positively associated with WASH and that this acts indirectly on WHZ as well. The objective of this study is to assess the causal change in WHZ by SES and WASH.

## Materials and methods

### Participants

Data for 10,478 children under 5 years old were collected through the Food Security Nutritional Surveillance Project (FSNSP) [44] in Bangladesh between February to November 2013. FSNSP followed a repeated cross-sectional design and targeted whole Bangladesh, dividing it into six zones vulnerable to malnutrition and food insecurity (coastal belt, eastern hills, *haor* region, *padma* chars, northern *chars* and the northwest flood plains) and seven divisional zones (Dhaka, Chittagong, Rajshahi, Barisal, Khulna, Sylhet and Rangpur). Household data were collected every four months (February 2013 to November 2013), covering three major seasons in Bangladesh: the post-aman harvest (January-April), the height of monsoon (May-August), and the post-aus harvest (September-December).

### Latent constructs

#### Socio-economic variables

A household socio-economic status (SES) latent variable was constructed from: ownership of homestead land, agricultural land, household presence of electricity, solar panel, radio/television, telephone/mobile phone, ceiling fan, *khat/chawki* (bed/cot), almirah (wardrobe)/showcase, refrigerator, table/chair, clock/watch, bicycle, motorcycle/scooter/tempo (automatic tricycle), animal drawn cart, car/truck, country boat, motorized boat, rickshaw/van, power tiller, shallow machine (primarily used as water pump), Uninterrupted Power Supply device (UPS)/electric generator, fishing net; cane/palm/trunks, dirt, bamboo with mud, tin, cement and bricks and planks/shingles as major construction material of house’s walls; wood, thatch/palm leaf and tin as major construction material of the roof; wood, straw/grass/leaves/agricultural waste, animal dung and other materials (kerosene/charcoal/bio-gas/natural gas/LPG gas etc.) as fuel used while cooking; ownership of cow/buffalo, sheep/goat/pig, chickens/duck/geese and small game (rabbits, pigeons, etc.). Additionally, food security status of the household (whether or not the house was food secure) as indicated by Household Food Insecurity Access Scale (HFIAS) [45]; income generating status of the mother of the child; education status of the mother (at least one year of formal schooling); and household income (previous month’s income).

#### Water supply, sanitation and hygiene (WASH)

The latent WASH variable was constructed using responses from mothers on the use of soap while washing clothes, child’s bottom, child’s hand and washing own hand after defecating, after cleaning a child, before feeding a child, before preparing food and before eating and during bathing (child and self). Other variables included were the use of hygienic latrine by household members and availability of a safe source of water for drinking. Safe water sources included rain or tubewell water and if it was covered stored for later use.

#### Covariates

Three covariates- child’s age, sex and residence type were adjusted for. Child’s age was regressed on WHZ only; child’s sex was regressed on WHZ and WASH; residence type (urban/rural) was regressed on SES, WASH and WHZ.

#### Anthropometric measurements

The weight of children was measured to the nearest 0.1 kg using a portable electronic weighing scale (TANITA Corporation Japan, model HD-305). Recumbent length of children less than two years of age and height for children aged between 2 to 5 years was measured to the nearest 0.1 cm using a locally made height and length board. All anthropometric measurements were performed following WHO guidelines [46]. WHZ was calculated according to the WHO growth standards [1].

#### Data analysis

Exploratory Factor Analysis (EFA) was used to calculate the factor loading values of the measured SES variables onto the latent SES construct. Variables with strong loading value (> 0.5) were considered for the final latent construct of SES in order to have a solid factor [47]. Variables that were used for the final SES construct were: presence of electricity in the household, possession of radio/television, ceiling fan, *almirah* (wardrobe), refrigerator, clock/watch, major constructing materials of a household, household food security status and monthly income. Measurement model technique was not applied for constructing the latent variable WASH as the number of variables was limited and all the variables used were included.

**Fig 1:**
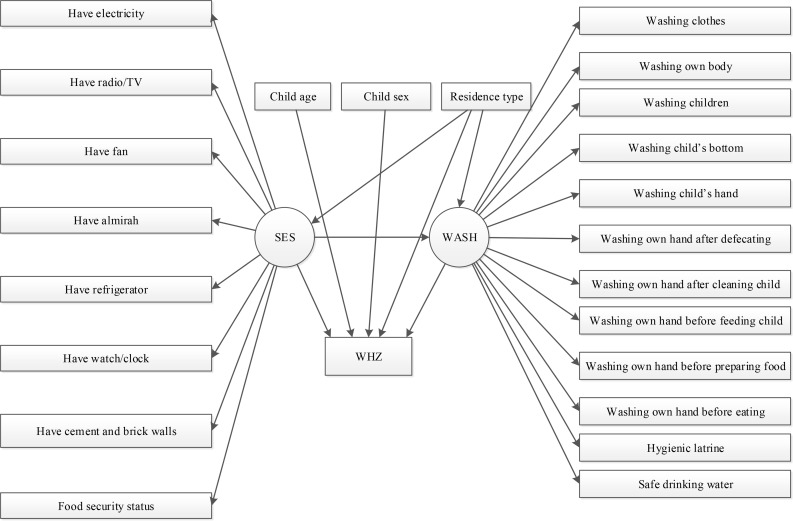
Conceptual model of causal pathways between SES, WASH and WHZ.

An expert-defined SEM was then constructed to evaluate (Fig 1): (i) whether SES has any direct statistically association with WHZ; (ii) whether WASH itself has any direct association with WHZ; and (iii) whether WASH mediates the relationship between SES and WHZ. The measurement component (i.e. the description of the latent variables) of the model included the WASH and SES constructs, whilst the structural component of the model included a direct pathway from SES to WHZ and an indirect pathway from SES to WHZ via the mediator latent variable WASH. The model also included child’s age, sex and location of residence (rural/urban) to control for their effects on WHZ and SES (excluding age given that household SES was assumed to be time invariant).

Reliability of the loadings on the latent variables was assessed using Chonbach’s alpha. Model fit was assessed using standard SEM diagnostics (chi-square, Root Mean Squared Error of Approximation (RMSEA), Comparative Fit Index (CFI) and Bentler-Bonett Nonnormed Fit (NNFI)). Standardized factor loading values (coefficients) with 95% CI along with p-value are reported.

All data were analyzed using STATA 14 (StataCorp. TX. USA).

### Ethical consideration and consent procedure

This study protocol titled “The Food Security Nutritional Surveillance Project (FSNSP): Secondary Data Analysis” was approved by the icddr,b research and ethical review committees. The FSNSP surveillance itself obtained ethical clearance from the European Union (Study number FOOD/2008/145-720). Verbal informed consent was taken from the FSNSP study participants. In case of children, consent was taken from the mother or primary caregiver. Written consent was not taken in order to avoid the stigma associated with signing paper documents especially in rural areas of Bangladesh. The outcome of the consent procedure was recorded in the consent form by the interviewer.

## Results

### Descriptive statistics

Among 10,478 children, the mean WHZ was -0.79 and the prevalence of wasting was 10.4% (95% CI: 9.84%, 11.0%). Over half (52%) of children included were male and mean age of the study children was 29.7 months. Around 92% of the study population was from the rural area and the average number of family members was 5.4. Twenty-six percent households had monthly income more than 10,000 Taka (approximately 128 USD). Day laboring was the main profession for majority of the main earners (42.6%). Nearly one fifth of mothers were involved in income generating activities and around 18% mothers had no formal schooling.

Soap was mostly used during washing clothes (81.7%), washing respondent’s own body (87.6%) and while washing own hands after defecation (55.9%). Prevalence of hand washing with soap was 1.6% before feeding a child, 4.4% before preparing food for the family and 4.9% before eating. Use of soap while washing children, while washing child’s bottom, while washing child’s hand and while washing own hand after cleaning the child were reported by 21%, 11.8%, 3.4% and 18.2% mothers/caretakers respectively. Use of a hygienic latrine was found in 15.5% households.

Half of the study population had government provided electricity in their households and 9% reported to use personal solar panels. Thirty-three percent of the households had television or radio and 83% households had mobile phone or telephone. Around 45% households had tin as construction material of external wall. Eighty-nine percent households had tin as construction material of the roof. The main construction material of the floor was sand/clay for majority of the household (80.4%). Wood (41.3%) and grass/straw (42.3%) were used as cooking fuel in most of the households. Tube well was the chief source of drinking water for majority (91%) of the families and around 84% families used safe water for drinking (tube well or rain water and covered if stored). Nearly two third (62%) of the families were found to be food secure. All other descriptive statistics are mentioned in Table 1.

**Table 1:** Descriptive statistics.

Indicators (N)		n	% (95% CI)
**Child sex (10478)**			
	Female	5117	48.8 (47.9, 49.8)
	Male	5361	51.2 (50.2, 52.1)
**Residence type (10478)**			
	Rural	9597	91.6 (91, 92.1)
	Urban	881	8.4 (7.9, 9)
Mother involved in income generating activity (9240)		1734	18.8 (18, 19.6)
Mother with no formal schooling (10478)		1910	18.2 (17.5, 19)
Household income above 10000 Tk (10478)		2739	26.1 (25.3, 27)
Household asset*			
Electricity		5220	49.8 (48.9–50.8)
Solar Panel		966	9.2 (8.7–9.8)
Radio/TV		3457	33 (32.1–33.9)
Telephone/Mobile phone		8752	83.5 (82.8–84.2)
Fan		5026	48 (47–48.9)
*Khat/chawki*		9792	93.5 (93–93.9)
Almirah		6325	60.4 (59.4–61.3)
Refrigerator		1101	10.5 (9.9–11.1)
Table/chair		8108	77.4 (76.6–78.2)
Watch/Clock		3964	37.8 (36.9–38.8)
Bicycle		2594	24.8 (23.9–25.6)
Motorcycle/scooter/tempo		738	7 (6.6–7.5)
Animal drawn cart		41	0.4 (0.3–0.5)
Car/truck		56	0.5 (0.4–0.7)
Country boat		421	4 (3.7–4.4)
Boat with Motor		115	1.1 (0.9–1.3)
Rickshaw/ van		754	7.2 (6.7–7.7)
Power Tiller		238	2.3 (2–2.6)
Shallow machine		739	7.1 (6.6–7.6)
UPS/Electric generator		86	0.8 (0.7–1)
Fishing net		2532	24.2 (23.4–25)
External wall material			
No walls		12	0.1 (0.1–0.2)
Cane/palm/trunks		761	7.3 (6.8–7.8)
Dirt		1326	12.7 (12–13.3)
Bamboo with mud		1448	13.8 (13.2–14.5)
Stone with mud		2	0 (0–0.1)
Plywood		6	0.1 (0–0.1)
Cardboard/polythin		17	0.2 (0.1–0.3)
Tin		4707	44.9 (44–45.9)
Cement & Bricks		2057	19.6 (18.9–20.4)
Stone with lime/cement		5	0 (0–0.1)
Wood planks/shingles		137	1.3 (1.1–1.5)
**Main roof material**			
No roof		1	0 (0–0.1)
Thatch palm leaf		398	3.8 (3.4–4.2)
Bamboo with mud		45	0.4 (0.3–0.6)
Cardboard/polythin		55	0.5 (0.4–0.7)
Tin		9368	89.4 (88.8–90)
Wood		12	0.1 (0.1–0.2)
Cement & bricks		584	5.6 (5.2–6)
Other		15	0.1 (0.1–0.2)
**Flooring material**			
Earth/sand		8425	80.4 (79.6–81.2)
Wood planks		75	0.7 (0.6–0.9)
Palm/Bamboo		107	1 (0.8–1.2)
Polished wood		28	0.3 (0.2–0.4)
Ceramic tiles		1834	17.5 (16.8–18.2)
Cement		8	0.1 (0–0.2)
**Cooking fuel**			
Wood		4325	41.3 (40.3–42.2)
Straw/ grass/agri. Waste		4434	42.3 (41.4–43.3)
Animal dung (wood)		1348	12.9 (12.2–13.5)
Others (Electricity/LPG/Piped Natural		371	3.5 (3.2–3.9)
**Source of drinking water**			0 (0–0)
Tubewell		9535	91 (90.4–91.5)
Piped Water		322	3.1 (2.8–3.4)
Dug Protected		15	0.1 (0.1–0.2)
Others (Surface water)		606	5.8 (5.4–6.2)
**Indicators**		**Mean (95% CI)**	
Wight for height z-score		-0.79 (-0.81, -0.77)	
Age of the child (months)		29.7 (29.37, 30.02)	
Presence of homestead land		6.75 (6.37, 7.13)	
Presence of agricultural land		46.75 (44.05, 49.44)	

*multiple response

### Measurement model and latent variables

The factor loading values for the observed SES variables were truncated at 0.5 during exploratory factor analysis. Eight variables out of 34 variables were retained for the latent variable SES: ‘household food insecurity’ and households to ‘have electricity’, ‘have radio/TV’, ‘have fan’, ‘have almirah’, ‘have refrigerator’, ‘have watch/clock’and ‘have brick and cement walls’. The factor loading values are mentioned in Table 2.

**Table 2:** Factor loading values for SES.

Variable used to construct SES	Factor1 (SES)
have fan	0.7394
have TV	0.6701
have electricity	0.6504
have almirah	0.5901
have cement and brick walls	0.5867
have watch clock	0.5310
food security status	0.5213
have refrigerator	0.5120
**Variables not used to construct SES**	
household income per month	0.4692
have table/chair	0.4672
have telephone/mobile phone	0.4252
maternal education status	0.3695
have motorcycle/scooter	0.3516
use grass/agricultural products as fuel	-0.3410
amount of agricultural land	0.3353
have bed/cot	0.3085
have cane/palm/trunks walls	-0.3031
use wood as fuel	0.2617
have bamboo with mud walls	-0.2382
amount of household land	0.2318
have 'shallow' water pumping machine	0.1925
have power tiller	0.1497
have rabbits	0.1293
have electric generator/uninterrupted power supply	0.1277
have car/truck	0.1235
maternal occupation status	-0.1231
have cow	0.1215
have chicken	0.108
have tin walls	-0.0913
have dirt walls	-0.0830
have fishing net	0.0594
use animal dung as fuel	-0.0377
have sheep	0.0337
have animal drawn cart	0.0307
have wooden roof	0.0233
have plank/shingle walls	-0.0207
have country boat	-0.0194
have motor boat	0.0139
have solar panel	-0.0010
use electricity/LPG/bio-gas as fuel	0.276
have bicycle	0.2744
have thatch palm leaf roof	-0.2339
have rickshaw/van	-0.0478

**Fig 2:**
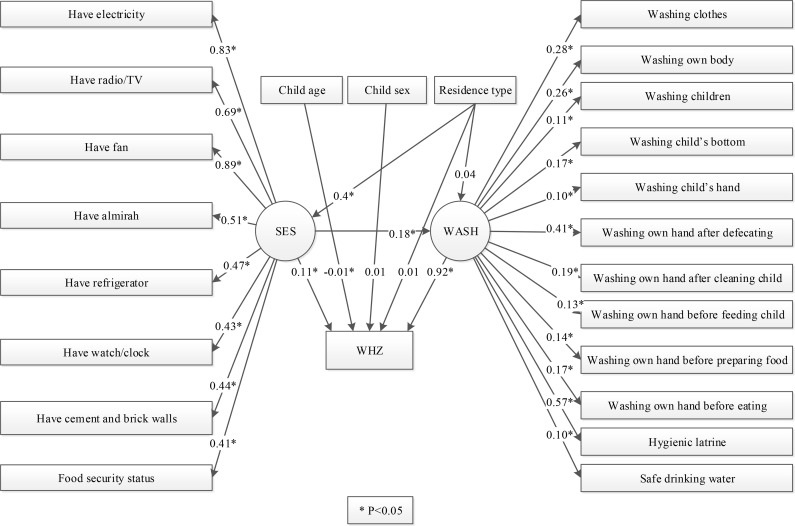
Structural Equation Model: Direct and Indirect Effects.

The eigenvalue for the 1^st^ factor was 4.78 and the proportion of variance it explained was 28%. The agreement (alpha) value of the eight variables was 0.817. All thirteen water, sanitation and hygiene variables were used to construct the latent WASH variable as we found all of them to be critical to define WASH. All standardized coefficient values of the observed variable on SES and WASH respectively and p-value are shown in Fig 2.

### Structural component

The structural model confirms that WHZ is significantly and positively affected by the two latent variable SES (p<0.05) and WASH (p<0.001) and the observed variable ‘child’s age’ (p<0.001) (Fig 2). WASH is also positively and significantly affected by SES (p<0.001) and type of residence (p<0.001). SES is also positively and significantly affected by types of residence (p<0.001). There was no evidence for an effect of child’s sex (p = 0.539) and types of residence (p = 0.700) on WHZ or child’s sex on WASH (p = 0.730).

When the effects in the SEM were decomposed into their direct and indirect associations, WHZ score was directly, significantly and positively affected by both SES and WASH (mean effect 0.5, p = 0.023; and 0.10, p<0.001 respectively). SES had a direct, positive and significant association with WASH (0.69, p<0.001). In terms of the hypothesized indirect pathway between SES and WHZ that was mediated by WASH, SES had an additional 0.07 average impact on WHZ (p<0.001).

The chi squared value of 9557.9 was statistically significant (p<0.001) with 244 degrees of freedom, RMSEA value of 0.06 (95% CI: 0.059–0.061), CFI value of 0.764 and TLI value of 0.736 indicated reasonably good fit of the full model. Moreover, the CD value suggests that the model explained 11.8% of the variance in WHZ.

The proportion of the effect of SES on WHZ mediated by WASH is approximately 60% (calculation not shown).

## Discussion

The relationship between SES and wasting has long been scrutinized, however the interrelationship between SES and WASH has often been ignored despite WASH representing an actionable target to reduce the prevalence of wasting. Here, we explicitly examining the interrelationship between these three variables. The SEM model showed that SES had statistically significant direct effect on WHZ, but crucially that there is a substantial indirect relationship that is mediated through WASH practices.

Our finding suggests that approximately 60% of the association between SES and the WHZ is mediated though WASH. This framework has been used to hypothesize that unimproved sanitation and failure to wash hand after defecation cause fecal contamination of home surroundings, which leads to fecal ingestion and contamination causing enteropathy [29], and ultimately results in childhood wasting. Similarly, contaminated water risks ingestion of fecal material more directly [12].

Participant’s compliance or motivation to follow WASH intervention protocols is couched in terms of their socio-economic circumstances along with their personal and communal belief, education status, perceived need, previous experience and visibility of the program’s impact [48]. Acting on WASH cannot be sustainably achieved without reference to the SES context [30], though equally projects to improve sanitation do not necessarily permeate to the level of behavioral practice in the home.

Two prior studies [17,32] found no evidence for SES or WASH impacts on wasting respectively, however neither examined the other driver or indeed their relationships. A secondary analysis of Bangladesh Demographic and Health Survey data of 2007, however found an independent association of maternal education but not socio-economic status with wasting [4]. Maternal education has been, however, a constituent part of defining SES in other studies [49] and may capture a wider set of conditions that shares variance with other SES metrics.

In a global context, evaluation of a three-year water treatment and intervention in rural Guatemala which assessed many handwashing practices that are common to our analysis along with safe water supply and hygienic latrine use, did not find any association between WASH interventions and WHZ from 929 under 5 children [25]. However, improved hand washing was found to be positively and significantly associated with WHZ during initial months but found to be diminished later on among Nepalese children [27].

Previous studies have included proxy indicators and used multiple regression to quantify the independent contribution of each variable to wasting. Such analyses fail to capture the multifaceted nature of SES and WASH domains or to address the multicollinearity of multiple indicators of the same theme. In contrast, the use of SEM deliberately represents WASH and SES as constructs of many components and allows explicit testing of their structural relationships. Additionally, regression of possible confounders such as age, sex and strata in the analysis reduced bias in the expression of the independent effect of the variables of interest.

In concordance with our hypothesis, findings from a Nepalese study [27] suggested that optimal hand washing practices can be a necessity for good health of the population living in crowded and contaminated areas but nonetheless, it cannot inhibit subclinical infections which is associated with child malnutrition. The investigators concluded that the ultimate factor adversely affecting child nutrition status is poor living conditions associated with poverty. In order for WASH interventions to sustain and have a significant impact on childhood recurrent infections, it is necessary to address the underlying poor socio-economic condition.

## Conclusion

In Bangladesh, the reductions in the prevalence of wasting has stalled despite dramatic early progress. Here we show that WASH factors account for 60% of the association between SES and wasting. Improvements to SES may result in sustainable reductions in the prevalence of wasting, but these are likely to be achieved through the implementation of WASH initiatives. We expect our findings to enhance the existing evidence base for the efficacy and effectiveness of WASH interventions. Translation of our findings might contribute to policy formulation in terms of justifying the inclusion of WASH components in nutrition and livelihood improvement strategies. Moreover, research uptake by the stakeholders in nutrition may aid in designing *value-for-money* and effective interventions for preventing child undernutrition especially wasting in Bangladesh.

## Limitations

It should be acknowledged that the data collection team for surveillance could not visit very remote hard-to-reach areas and therefore data were not collected from some hard to reach areas. Much of the information was collected through maternal responses which might have introduced recall bias. No handwashing demonstration was observed. The data analysis was constrained by the number of variables originally selected for the food security and nutrition survey. The data is country specific and therefore special consideration must be taken on extrapolating the findings to other contexts.

## Supporting information

S1 File STATA_Log. (SMCL)

S1 Dataset Data_Set. (XLS)

## References

[1] WHO Multicentre Growth Reference Study Group (2006) WHO Child Growth Standards: Length/height-for-age, weight-for-age, weight-for-length, weight-for-height and body mass index-for-age: Methods and development. Geneva.

[2] Bloss E, Wainaina F, Bailey RC (2004) Prevalence and predictors of underweight, stunting, and wasting among children aged 5 and under in western Kenya. Journal of tropical pediatrics 50: 260–270. 15510756 10.1093/tropej/50.5.260

[3] World Health Organization (2015) Child growth indicators and their interpretation. WHO | Global Database on Child Growth and Malnutrition

[4] Siddiqi MNA, Haque MN, Goni MA (2011) Malnutrition of under-five children: evidence from Bangladesh. Asian Journal of Medical Sciences 2: 113–119.

[5] Wang Y, Moreno LA, Caballero B, Cole TJ (2006) Limitations of the current World Health Organization growth references for children and adolescents. Food & Nutrition Bulletin 27: 175–188. 10.1177/15648265060274S502 17361655

[6] Fuchs C, Sultana T, Ahmed T, Iqbal Hossain M (2014) Factors associated with acute malnutrition among children admitted to a diarrhoea treatment facility in Bangladesh. International journal of pediatrics 2014. 10.1155/2014/267806 PMC3964844 24734048

[7] Kennedy E, Branca F, Webb P, Bhutta Z, Brown R (2015) Setting the scene: An overview of issues related to policies and programs for moderate and severe acute malnutrition. Food & Nutrition Bulletin 36: 9S–14S. 10.1177/15648265150361S102 25902609

[8] Manary MJ, Sandige HL (2008) Management of acute moderate and severe childhood malnutrition. Bmj 337. 10.1136/bmj.a2180 19008271

[9] Schofield C, Ashworth A (1996) Why have mortality rates for severe malnutrition remained so high? Bulletin of the World Health Organization 74: 223 8706239 PMC2486901

[10] Ahmed T, Ali M, Ullah MM, Choudhury IA, Haque ME, et al (1999) Mortality in severely malnourished children with diarrhoea and use of a standardised management protocol. The Lancet 353: 1919–1922. 10.1016/S0140-6736(98)07499-6 10371570

[11] Shekar M, Heaver R, Lee Y-K (2006) Repositioning nutrition as central to development: A strategy for large scale action: World Bank Publications.

[12] Dangour AD, Watson L, Cumming O, Boisson S, Che Y, et al (2013) Interventions to improve water quality and supply, sanitation and hygiene practices, and their effects on the nutritional status of children. Cochrane Database Syst Rev 8. 10.1002/14651858.CD009382.pub2 PMC11608819 23904195

[13] Gross R, Webb P (2006) Wasting time for wasted children: severe child undernutrition must be resolved in non-emergency settings. The Lancet 367: 1209–1211. 10.1016/S0140-6736(06)68509-7 16616563

[14] Rayhan MI, Khan MSH (2006) Factors causing malnutrition among under five children in Bangladesh. Pak J Nutr 5: 558–562.

[15] Smith LC, Ruel MT, Ndiaye A (2005) Why is child malnutrition lower in urban than in rural areas? Evidence from 36 developing countries. World Development 33: 1285–1305.

[16] Pelletier D (2002) Toward a common understanding of malnutrition: assessing the contributions of the UNICEF framework. World Bank/UNICEF Nutrition Assessment.

[17] Mohsena M, Mascie-Taylor C, Goto R (2010) Association between socio-economic status and childhood undernutrition in Bangladesh; a comparison of possession score and poverty index. Public health nutrition 13: 1498–1504. 10.1017/S1368980010001758 20576197

[18] Frozanfar MK, Yoshida Y, Yamamoto E, Reyer JA, Dalil S, et al (2016) Acute malnutrition among under-five children in Faryab, Afghanistan: prevalence and causes. Nagoya journal of medical science 78: 41 27019527 PMC4767513

[19] de Pee S, Grais R, Fenn B, Brown R, Briend A, et al (2015) Prevention of acute malnutrition: distribution of special nutritious foods and cash, and addressing underlying causes—what to recommend when, where, for whom, and how. Food and nutrition bulletin 36: S24–S29. 10.1177/15648265150361S104 25902611

[20] Sharma K (2012) Malnutrition in children aged 6–59 months in Mugu district. Journal of Nepal Health Research Council. 23034380

[21] Imdad A, Sadiq K, Bhutta ZA (2011) Evidence-based prevention of childhood malnutrition. Current Opinion in Clinical Nutrition & Metabolic Care 14: 276–285. 21415736 10.1097/MCO.0b013e328345364a

[22] Gwatkin DR, Rutstein S, Johnson K, Pande R, Wagstaff A (2000) Socio-economic differences in health, nutrition, and population. Washington, DC: World Bank. 18293634

[23] Waddington H, Snilstveit B, White H, Fewtrell L (2009) Water, sanitation and hygiene interventions to combat childhood diarrhoea in developing countries. New Delhi: International Initiative for Impact Evaluation.

[24] Banda K, Sarkar R, Gopal S, Govindarajan J, Harijan BB, et al (2007) Water handling, sanitation and defecation practices in rural southern India: a knowledge, attitudes and practices study. Transactions of the royal society of tropical medicine and hygiene 101: 1124–1130. 10.1016/j.trstmh.2007.05.004 17765275

[25] Arnold B, Arana B, Mäusezahl D, Hubbard A, Colford JM (2009) Evaluation of a pre-existing, 3-year household water treatment and handwashing intervention in rural Guatemala. International Journal of Epidemiology 38: 1651–1661. 10.1093/ije/dyp241 19574492 PMC2786251

[26] Arnold BF, Null C, Luby SP, Unicomb L, Stewart CP, et al (2013) Cluster-randomised controlled trials of individual and combined water, sanitation, hygiene and nutritional interventions in rural Bangladesh and Kenya: the WASH Benefits study design and rationale. BMJ open 3: e003476 10.1136/bmjopen-2013-003476 23996605 PMC3758977

[27] Langford R, Lunn P, Brick CP (2011) Hand‐washing, subclinical infections, and growth: A longitudinal evaluation of an intervention in Nepali slums. American Journal of Human Biology 23: 621–629. 10.1002/ajhb.21189 21630368

[28] Luby SP, Agboatwalla M, Painter J, Altaf A, Billhimer WL, et al (2004) Effect of intensive handwashing promotion on childhood diarrhea in high-risk communities in Pakistan: a randomized controlled trial. Jama 291: 2547–2554. 10.1001/jama.291.21.2547 15173145

[29] Ngure FM, Reid BM, Humphrey JH, Mbuya MN, Pelto G, et al (2014) Water, sanitation, and hygiene (WASH), environmental enteropathy, nutrition, and early child development: making the links. Annals of the New York Academy of Sciences 1308: 118–128. 10.1111/nyas.12330 24571214

[30] Isunju J, Schwartz K, Schouten M, Johnson W, van Dijk MP (2011) Socio-economic aspects of improved sanitation in slums: a review. Public health 125: 368–376. 10.1016/j.puhe.2011.03.008 21616514

[31] Humphrey JH (2009) Child undernutrition, tropical enteropathy, toilets, and handwashing. The Lancet 374: 1032–1035. 10.1016/S0140-6736(09)60950-8 19766883

[32] Lin A, Arnold BF, Afreen S, Goto R, Huda TMN, et al (2013) Household environmental conditions are associated with enteropathy and impaired growth in rural Bangladesh. The American journal of tropical medicine and hygiene 89: 130–137. 10.4269/ajtmh.12-0629 23629931 PMC3748469

[33] Luby SP, Halder AK, Huda T, Unicomb L, Johnston RB (2011) The effect of handwashing at recommended times with water alone and with soap on child diarrhea in rural Bangladesh: an observational study. PLoS medicine 8: 798. 10.1371/journal.pmed.1001052 PMC3125291 21738452

[34] Ehiri J, Ejere H (2003) Hand washing for preventing diarrhoea. The Cochrane Library. 10.1002/14651858.CD004265.pub2 18254044

[35] Walker JE (1925) The germicidal properties of soap. Journal of Infectious Diseases 37: 181–192.

[36] Mittermayer H, Rotter M (1975) [Comparative investigations on the efficacy of tap water, some detergents and ethanol on the transient flora of the hands (author's transl)]. Zentralblatt fur Bakteriologie, Parasitenkunde, Infektionskrankheiten und Hygiene Erste Abteilung Originale Reihe B: Hygiene, praventive Medizin 160: 163–172. 1099852

[37] Lowbury E, Lilly H, Bull J (1964) Disinfection of hands: removal of transient organisms. British medical journal 2: 230–233. 14153905 10.1136/bmj.2.5403.230 PMC1817850

[38] Vidal M, Kruger E, Durán C, Lagos R, Levine M, et al (2005) Single multiplex PCR assay to identify simultaneously the six categories of diarrheagenic Escherichia coli associated with enteric infections. Journal of clinical microbiology 43: 5362–5365. 10.1128/JCM.43.10.5362-5365.2005 16208019 PMC1248459

[39] Black R, Merson M, Rahman AM, Yunus M, Alim AA, et al (1980) A two-year study of bacterial, viral, and parasitic agents associated with diarrhea in rural Bangladesh. Journal of infectious diseases 142: 660–664. 6257795 10.1093/infdis/142.5.660 PMC7109920

[40] Khan NZ, Ferdous S, Islam R, Sultana A, Durkin M, et al (2009) Behaviour problems in young children in rural Bangladesh. Journal of tropical pediatrics 55: 177–182. 10.1093/tropej/fmn108 19066172

[41] VanderWeele TJ (2012) Invited commentary: structural equation models and epidemiologic analysis. American journal of epidemiology: kws 213. 10.1093/aje/kws213 PMC3530375 22956513

[42] Gunzler D, Chen T, Wu P, Zhang H (2013) Introduction to mediation analysis with structural equation modeling. Shanghai archives of psychiatry 25: 390 10.3969/j.issn.1002-0829.2013.06.009 24991183 PMC4054581

[43] Chavance M, Escolano S, Romon M, Basdevant A, de Lauzon-Guillain B, et al (2010) Latent variables and structural equation models for longitudinal relationships: an illustration in nutritional epidemiology. BMC medical research methodology 10: 37 10.1186/1471-2288-10-37 20433707 PMC2873513

[44] Helen Keller International B (2014) FSNSP History. Food Security Nutritional Surveillance Project 2014.

[45] Coates J, Swindale A, Bilinsky P (2007) Household Food Insecurity Access Scale (HFIAS) for measurement of food access: indicator guide. Washington, DC: Food and Nutrition Technical Assistance Project, Academy for Educational Development.

[46] Cogill B (2003) Anthropometric indicators measurement guide.

[47] Osborne JW, Costello AB (2009) Best practices in exploratory factor analysis: Four recommendations for getting the most from your analysis. Pan-Pacific Management Review 12: 131–146.

[48] du Preez M, Conroy RM, Ligondo S, Hennessy J, Elmore-Meegan M, et al (2011) Randomized intervention study of solar disinfection of drinking water in the prevention of dysentery in Kenyan children aged under 5 years. Environmental science & technology 45: 9315–9323. 21936492 10.1021/es2018835

[49] Psaki SR, Seidman JC, Miller M, Gottlieb M, Bhutta ZA, et al (2014) Measuring socioeconomic status in multicountry studies: results from the eight-country MAL-ED study. Population health metrics 12: 1. 24656134 10.1186/1478-7954-12-8 PMC4234146

