# Correction: Examining the relationship between socio-economic status, WASH practices and wasting

**DOI:** 10.1371/journal.pone.0226294

**Published:** 2019-12-04

**Authors:** The PLOS ONE Editors

After publication of this article [1], concerns were raised about this article. Specifically, the following:

1. The article does not present a clearly-articulated justification for the study and an overview of the problem addressed by the study.
2. The survey sampling procedures are not described in detail.
3. The unit of observation considered in the analyses, and whether the data are being treated as longitudinal or cross-sectional, is not clear.
4. It is not clear whether changes were made to the SEM model structure between the original conceptual model and the final model.
5. The approach used for estimation of loadings in the SEM is not clear.
6. The relationship between path coefficient values and *p* values shown in Fig 2 and reported in the Results are not clear. In addition, the method used to calculate the portion of the effect of SES on WHZ mediated by WASH is not clear.
7. The meaning of the abbreviation ‘CD’ is not given.
8. It is not clear whether the exogenous variables child age, sex, and residence type are nontrivially inter-correlated.

The authors have responded to these concerns by providing the following additional information:

1. Enteric infections leading to growth faltering and other forms of undernutrition prevail among Bangladeshi children and elsewhere. Optimal WASH practices have been found to reduce the burden of enteric infections. The critical role of optimal WASH practices for better health is well established and improved water, sanitation and hygiene were identified as necessary conditions to prevent the more acute form of undernutrition- wasting. WASH studies conducted in Bangladesh in the context of child undernutrition are limited. The study was thus warranted to understand the role of WASH in Bangladesh in terms of association with acute malnutrition in children. Moreover, the statistical method used in this study can show direction of the effect and thus the inferences drawn are more robust than that from multiple linear regression.
  WASH in this study reflects the optimal water, sanitation and hygiene practices of the surveyed households and encompasses variables reflecting the use of soap by mother while washing clothes, child’s bottom, child’s hand and washing own hand after defecating, after cleaning a child, before feeding a child, before preparing food and before eating and during bathing (child and self). Other variables included were the use of hygienic latrine by household members and availability of a safe source of water for drinking. Safe water sources included rain or tube-well water and if it was covered stored for later use.
2. In terms of the survey sampling procedure, the country is divided into 13 areas or *strata*, of those, six are agro-ecologically based zones and the remaining are the seven administrative divisions. The primary sampling unit (PSU) was *upazila* or sub-district. From each agro-economic zone 12 *upazilas* were selected with replacement by rotation whereas 22 *upazilas* were selected from the rest with replacement but without rotation and stratified by divisions. The secondary sampling unit (SSU) was *mohalla* or village. Households in all surveyed *upazilas* were divided into equal sized clusters and four clusters were randomly selected from each *upazila*. Every time four additional clusters were selected if the *upazila* was selected more than once. Thereafter, every 3^rd^ household in Chittagong Hill Tracts was selected and for the rest of the country, every 4^th^ household was selected. The entry direction to a village was random and the 1^st^ house in the north, east, south and west was selected and then household selection was counter-clockwise.
3. As regards the unit of observation, the unit is the child and not more than one record per child was analyzed. The data used in this study has been treated as cross-sectional and there is one record per child. Therefore, the data is not longitudinal or repeated measure.
4. In terms of the model structure, some changes were made to the SES construct. Depending on the factor loading values of the exploratory factor analysis, only eight variables out of 34 variables were retained.
5. Regarding loading analysis, we truncated the loading value at a fixed value during EFA and during CFA the latent variables were standardized.
6. The values reported in Fig 2 are coefficients of corresponding equations and ‘*’ (asterisk) represent the p-value, while in the result section only p-values are reported. We have rechecked the p-values of the coefficients and they are significant. The proportion of the effect of SES on WHZ mediated by WASH is ((0.18 × 0.92)/ {0.11 + (0.18*0.92)) = 0.6 or approximately 60% of the total effect. The STATA software provides all three effects, direct, indirect and total. However, as our primary concern was to observe the effect of WASH on WHZ and effect of SES mediated by WASH on WHZ, we did not show all the effects available from the output.
7. The abbreviation ‘CD’ refers to the Coefficient of Determination.
8. The variables were not correlated with each other and so the inter-correlations were zero.
